# Feasibility and Safety of Absorbable Knotless Wound Closure Device in Laparoscopic Myomectomy

**DOI:** 10.1155/2016/2849476

**Published:** 2016-06-27

**Authors:** Chying-Chyuan Chan, Ching-Yu Lee

**Affiliations:** ^1^Department of Obstetrics and Gynecology, Taipei City Hospital, Zhongxiao Branch, Taipei 11556, Taiwan; ^2^Department of Obstetrics and Gynecology, Taipei City Hospital, Renai Branch, Taipei 10629, Taiwan; ^3^National Defense Medical Center, Taipei 114, Taiwan

## Abstract

*Purpose.* Myomectomy has been performed through laparoscopy. Suturing is known as rate-limiting step in laparoscopic myomectomy. The present study was aimed at comparing the clinical outcomes of absorbable knotless wound closure device with the results of conventional suturing.* Methods*. This prospective study included 62 women who underwent laparoscopic myomectomy at Taipei City Hospital, Zhongxiao Branch, from January 2010 through to August 2012. The patients were randomized into two groups according to suturing materials, the knotless group and the 2-0 Vicryl suture group. Patient demographics, overall operative time, and intraoperative blood loss were compared between two groups.* Results*. Demographic characteristics and laboratory variables before surgery were comparable. Operative time was significantly shorter in knotless group compared with that in 2-0 Vicryl suture group (112 ± 47 versus 147 ± 63 minutes; *p* < 0.05). The results revealed a significant difference in intraoperative blood loss between two groups (knotless versus 2-0 Vicryl: 112.8 ± 54.2 versus 143.6 ± 64.9). Use of absorbable knotless wound closure device was associated with greater hemostasis compared with that of 2-0 Vicryl. During a 2-year follow-up period, 12 patients (46.2%) from the group with absorbable knotless wound closure device and 14 patients (38.9%) from 2-0 Vicryl suture group became pregnant.* Conclusion.* Closure of myometrium using absorbable knotless wound closure device after laparoscopic myomectomy resulted in a shorter operative time and less blood loss.

## 1. Introduction

Uterine fibroid is one of the most common neoplastic conditions of reproductive organs in women. It occurs in up to 30% of women of childbearing age [1, 2]. The incidence of uterine myoma increases with age until menopause. The majority of uterine fibroids are asymptomatic and require no treatment, which are discovered upon examinations for other complaints [3]. Symptomatic fibroids are associated with several morbidities including menstrual bleeding, iron deficiency anemia, pelvic pressure/pain, and infertility [4]. Treatment modalities indicated for women with symptomatic fibroids include hormone therapy, surgical procedures, and uterine artery embolization [5, 6]. Nevertheless, the treatments are provided by gynaecologist based on the practice setting and the preferences of the patients.

Laparoscopic myomectomy has been indicated for uterine fibroid patients who wish to preserve the uterus [7]. It offers several advantages over traditional laparotomy, including minimal blood loss, rapid recovery, reduced postoperative pain, fewer complications, shorter operating time, and satisfying cosmetic outcomes [8]. Due to the nature of laparoscopic technique, the surgery is associated with difficulties in quality suturing particularly knot tying that maintain adequate tension of the suture line [9]. Several modalities have been described to facilitate the suturing in open or laparoscopic procedures, including barded suture [10].

This study was aimed at examining the feasibility of use of absorbable knotless wound closure device in the closure of myometrium after laparoscopic myomectomy. We compared the surgical results after laparoscopic myomectomy enucleation of myomas incorporating with absorbable knotless wound closure device and conventional suture.

## 2. Materials and Methods

### 2.1. Patients

Patients who were diagnosed with uterine myomas and underwent laparoscopic myomectomy at Zhongxiao Campus, Taipei City Hospital, between January 2010 and August 2012 were eligible for inclusion. Patients were selected based on physical characteristics and ultrasonography findings prior to operation. Transvaginal ultrasound evaluation was carried out on day 5 or 6 after period to determine the size and shape of uterus. The criteria for inclusion were premenopausal woman with symptomatic myomas, the presence of at least one intramural myoma >4 cm or multiple myomas, and the absence of submucous myomas. Patients were excluded if they had undergone any concomitant surgical procedure to treat reproductive system diseases either before or after the laparoscopic myomectomy. Other exclusion criteria were menorrhagia and gonadotropin releasing hormone (GnRH) agonist treatment. Eligible patients were simply randomized blindly before surgical procedure by means of draw into two groups, namely, Group 1 receiving uterine laparoscopic myomectomy with absorbable knotless wound closure device (2-0 polydioxanone suture, PDS) and Group 2 receiving uterine laparoscopic myomectomy with the 2-0 Vicryl suture. The protocol of this prospective, randomized clinical study was approved by the Institutional Review Board of Taipei City Hospital. All participants signed and gave a written consent form prior to inclusion.

### 2.2. Surgical Procedures

All patients were operated on under general endotracheal anesthesia in a dorsal lithotomy and Trendelenburg position by the same experienced anesthesia team. A Foley catheter and a uterine manipulator were placed inside the bladder and the uterus. After filling abdominal cavity with carbon dioxide, three incisions were made in the lower abdomen, including one at the navel and the others below the bikini line. Laparoscope was placed through the incisions and utilized to confirm the location, size, number, maximum diameter, and type of myomas. All women received oxytocin diluted in saline (10 IU/mL) at a rate of 40 mU/min and an intramyometrial injection of Pitressin (10 IU/mL). A vertical incision of uterus was made into the serosal layer using Harmonic Scalpel (Ethicon Inc., Somerville, NJ) with preservation of two-thirds of pseudocapsule followed by enucleation of myomas. Hemostasis was maintained using LigaSure*™* Vessel Sealing Generator (Soma Technology Inc., Bloomfield, CT). After myomectomy, uterus was closed using double-layer suture technique. The suture was started at serosal layer of one end, passed through myometrial defects, and drawn through the serosal layer of the opposite end. In Group 1 (*n* = 26), closure of uterus was performed using continuous suturing with absorbable knotless wound closure device (2-0 PDS) placed with an interval of 1 cm using round needle. In Group 2, uterus was closed using interrupted suture technique with the 2-0 Vicryl sutures (1 cm apart) and round needle. All knots were tied extracorporeally and pushed inside with the aid of laparoscopic knot pusher. The enucleated myomas were extracted using electromechanical morcellator intracorporeally (Ethicon Inc., Somerville, NJ, USA). At the end of the operation, carbon dioxide was released and the incisions for laparoscope were closed with 4-0 Vicryl suture (Ethicon Inc., Somerville, NJ). All patients were postoperatively examined at 3 months for healing of the myomectomy scar using transvaginal ultrasonography.

### 2.3. Variables Compared

The variables compared between 2 groups included age, intraoperative blood loss which was calculated by subtracting the amount of physiological saline used for peritoneal washing from the total amount of fluid removed via suction, operation time that was measured as the time from the first incision to the time when the skin incision was closed, change in hemoglobin (ΔHb) that was determined by subtracting the Hb (g/dL) measured at 8 hours postoperatively from that measured prior to surgery, myoma mass, and change in hematocrit (ΔHt) that was analyzed by deducing the Ht (%) measured at 8 hours postoperatively from that measured prior to surgery.

### 2.4. Statistical Analysis

Data were analyzed by independent two-sample *t*-tests and were presented as mean ± SD. A probability (*p*) value of <0.05 was considered statistically significant. All statistical analyses were performed with SPSS statistical software (SPSS, Chicago, IL, USA).

## 3. Results

A total of 62 women with symptomatic uterine fibroids undergoing laparoscopic myomectomy were enrolled. All patients underwent laparoscopic myomectomy with general anesthesia. Of the patients, 26 had a uterine closure by absorbable knotless wound closure device (2-0 PDS), whereas 2-0 Vicryl suture was used on the others. As shown in Table 1, two groups were comparable with respect to age, body weight, and birth history. The results showed that preoperative complete blood count parameters, Hb and Ht, were comparable in both groups.

We next compared the clinical outcome of two different suturing techniques. The means of operation time were 112 minutes (±47 minutes) and 147 minutes (±63 minutes) in the knotless (2-0 PDS) groups and 2-0 Vicryl suture, respectively (*p* < 0.05; Table 2). The patient in the group with absorbable knotless wound closure device (2-0 PDS) exhibited significantly less intraoperative blood loss (112.8 ± 54.2 mL) compared with that of group with 2-0 Vicryl suture (143.6 ± 64.9 mL) (*p* < 0.05). Our data showed significant differences in changes in hemoglobin between two groups (*p* < 0.05) (Table 2). Change in hematocrit was significantly less in patients with absorbable knotless wound closure device (2-0 PDS) than in those with 2-0 Vicryl suture (4.2 ± 1.2% versus 6.6 ± 2.1%; *p* < 0.05). Intraoperative blood transfusion was made in 1 patient (3.8%) and 3 patients (8.3%) with absorbable knotless wound closure device (2-0 PDS) and 2-0 Vicryl suture, respectively. Postoperative 3-month transvaginal ultrasonography revealed uterine defect in 1 patient (3.8%) and 6 patients (16.7%) with absorbable knotless wound closure device (2-0 PDS) and 2-0 Vicryl suture, respectively (Table 2). No patient reported damage to other organs and exhibited postoperative complication. There was no patient requiring conversion to laparotomy.

During a 2-year follow-up period, 12 patients (46.2%) from the group with absorbable knotless wound closure device (2-0 PDS) and 14 patients (38.9%) from 2-0 Vicryl suture group became pregnant. Of the 26 patients, a patient (3.8%) in 2-0 Vicryl suture group had an abortion at the 24th week of gestation due to uterine rupture, whereas the others completed their pregnancy period and underwent Caesarean section.

## 4. Discussion

In the present study, we compared barbed suture device with conventional suture in a setting of laparoscopic myomectomy for uterine fibroids. We demonstrated that the laparoscopic suturing incorporating with absorbable knotless wound closure device exhibited a reduced operation time by approximately 24% compared with conventional sutures. We found that intraoperative blood loss was lower in the group using barbed suture. Absorbable knotless wound closure device provides favorable hemostasis during laparoscopic myomectomy.

Suturing is considered one of the difficult, tedious, and time-consuming procedures in laparoscopic surgery. Many suture materials and technologies have been introduced into laparoscopic surgeons' armamentarium. Use of barbed sutures has been shown to reduce suturing time for reapproximation of the myometrium [11]. In the present study, we demonstrated that use of barbed suture materials resulted in a significantly decreased overall operating time of laparoscopic myomectomy. Our finding is in agreement with a recent study in which reduction in operating time was significant in favor of V-Loc suture [12]. The reduced operation time is suggested to be attributed to less knot tying required. In addition, it is taken into account that the need for assistance to maintain suture line tension over procedure is unnecessary, resulting in accelerated suturing process. On the other hand, Alessandri et al. showed a significantly reduced suturing time in laparoscopic surgery with the use of V-Loc sutures, not operation time [11]. A recent study has shown that proficiency in passing the suture through the loop of the V-Loc suture is essential [13]. Besides suture material, pharmacological intervention is suggested to contribute to reduction in operating time. GnRH agonist has been suggested as a pretreatment to facilitate abdominal or laparoscopic myomectomy. In this study, the suturing method employed resulted in a significantly decreased operating time in patients not using GnRH agonist. Aoki et al. have reported reducing operating time using V-Loc sutures in subjects undergoing GnRH agonist treatment [12]. A meta-analysis study has shown that the overall operating time of laparoscopic myomectomy with or without preoperative GnRH agonist treatment was comparable [14]. It is likely that benefits of preoperative use of GnRH agonist are limited in aspect of laparoscopic myomectomy.

Laparoscopic myomectomy has been associated with favorable outcomes such as shorter hospital stay and less blood loss. Effect of barbed suture on hemostasis in laparoscopic myomectomy is controversial. Recent studies have reported that use of barbed suture was associated with significantly reduced blood loss [11, 13]. On the other hand, it has been reported that barbed suture had no effect on blood loss [12, 15]. In the present study, we found that use of absorbable knotless wound closure device led to a significantly reduced blood loss compared with that of conventional suturing. It is worth noting that vasopressin was administrated in the subjects of two studies which reported no difference in blood loss among groups. It is suggested that vasopressin contributes to hemostasis during laparoscopic surgeries. Given our findings, it is implied that use of barbed suture is effective in reducing intraoperative blood loss in absence of pharmacological interventions. In addition to blood loss, other studies have reported postoperative complications associated with barbed suture such as dehiscence and bleeding [16, 17]. It has been reported that barbed suture caused small bowel injury and obstruction [18–20]. Our findings showed that use of barbed suture in laparoscopic myomectomy is relatively safe, supported with previous studies [15, 17, 21].

Laparoscopic myomectomy is indicated in patients with fibroids who wish to preserve fertility. The surgical procedure is suggested to be safe and has no impact on pregnancy and delivery rate [22–24]. In this study, we compared the pregnancy outcomes of barbed suture and conventional suture materials used in laparoscopic myomectomy. We found that there was a nonstatistically significant trend towards higher pregnancy rates in Group 1. Nevertheless, our study was not sufficiently powered to determine whether or not this is a trend truly representing a difference. There was no uterine rupture during pregnancy in barbed suture groups, suggesting that use of barbed suture in laparoscopic myomectomy is safe. Kumakiri et al. have reported that, in 111 women after laparoscopic myomectomy, 79% completed vaginal delivery without uterine rupture [25]. However, the findings need to be examined further in a prospective controlled study with a larger population of patients.

There are several limitations in the present study. It is a single-surgeon and single-institution series and the results may not apply to other patient populations and other surgeons. This limitation is common in previous studies examining barbed suture in laparoscopic surgery. Secondly, this study has small sample size that may affect the power of the study and conclusions drawn from the data. However, this study consists of a control group for comparison, which included patients of similar age with similar fibroid characteristics. In addition, this study has short follow-up and lacked assessment of patient satisfaction, which merits a further study with longer follow-up.

## 5. Conclusion

Closure of myometrium using barbed suture after laparoscopic myomectomy resulted in a shorter operative time and less blood loss. The use of barbed sutures had no impact on pregnancy and delivery. The suture materials represent an opportunity for quality laparoscopic suturing with favorable outcomes.

## Figures and Tables

**Table 1:** Baseline characteristics of study participants.

Variables (mean ± SD)	Group 1 absorbable knotless wound closure device (*n* = 26)	Group 2 2-0 Vicryl (*n* = 36)	*p* value
Age (yr)	36.4 + 4.2	37.9 + 4.9	ns
Body weight (Kg)	58.9 + 8.2	56.1 + 9.7	ns
Birth history	1.4 + 0.8	1.1 + 0.9	ns
Preoperative Hb (g/dL)	12.9 + 1.2	12.7 + 1.4	ns
Preoperative Ht (%)	37.7 + 6.2	38.8 + 7.4	ns

SD: standard deviation; ns: not significant.

**Table 2:** Comparison of clinical outcomes of laparoscopic myomectomy with absorbable knotless wound closure device or 2-0 Vicryl suture.

	Group 1 absorbable knotless wound closure device (*n* = 26)	Group 2 2-0 Vicryl (*n* = 36)	*p* value
Myoma weight (g)	179.6 ± 53.2	152.5 ± 47.9	ns
Total operation time (min)	112 ± 47	147 ± 63	<0.05
Intraoperative blood loss (mL)	112.8 ± 54.2	143.6 ± 64.9	<0.05
ΔHb (g/dL)^a^	1.8 ± 1.2	2.2 ± 1.9	<0.05
ΔHt (%)^b^	4.2 ± 1.2	6.6 ± 2.1	<0.05
Intraoperative transfusion^c^	1 (3.8%)	3 (8.3%)	—
Uterine defect^d^	1 (3.8%)	6 (16.7%)	—

^a^Mean change in Hb value before operation and 8 hours after operation.

^b^Mean change in Ht value before operation and 8 hours after operation.

^c^Massive intraoperative bleeding over 500 mL or postoperative Hb value lower than 8 g/dL.

^d^Postoperative 3-month transvaginal ultrasound examination for uterine integrity/defect.
